# AL-Amyloidosis Presenting with Negative Congo Red Staining in the Setting of High Clinical Suspicion: A Case Report

**DOI:** 10.1155/2012/593460

**Published:** 2012-12-09

**Authors:** Kristina Bowen, Nina Shah, Matthew Lewin

**Affiliations:** ^1^Department of Internal Medicine, William Beaumont Army Medical Center, 5005 N Piedras Street, Building 7777, 9th floor East, El Paso, TX 79920, USA; ^2^Department of Nephrology, William Beaumont Army Medical Center, 5005 N Piedras Street, Building 7777, 12th floor, El Paso, TX 79920, USA; ^3^Propath Services, 1355 River Bend Drive, Dallas, TX 75247, USA

## Abstract

A histologic diagnosis of amyloidosis requires acquiring tissue containing amyloid fibrils from an affected organ or alternate site. The biopsy site and staining techniques may influence testing accuracy. We present a case in which systemic amyloidosis was suspected; however, biopsies of the bone marrow, an osteosclerotic bone lesion, arterial and venous vessels, and the fat pad were all negative for the diagnostic Congo red stain. An eventual renal biopsy demonstrated AL-amyloidosis, kappa light chain associated with extensive vascular interstitial, and glomerular, involvement. Choice of biopsy site, as well as staining and analysis of the tissue, can influence sensitivity and specificity of amyloid testing. Fat-pad biopsies are less invasive and offer reasonable sensitivity. Bone marrow samples are only diagnostic up to 63% of the time. A renal biopsy offers improved sensitivity and is generally safe in experienced hands, but is a more invasive procedure with increased number of relative contraindications and complications. The choice of the biopsy site should be based on considering the expected yield, accessibility of the site, and the risks associated with the procedure.

## 1. Introduction

Amyloidosis is the deposition of insoluble protein deposits either systemically or in a specific organ. Amyloidosis is classified by the type of protein into several categories including light-chain (AL), amyloid-associated protein (AA), or familial (AF). AL-type amyloidosis, known as primary amyloidosis, is the result of deposition of immunoglobulin light chains. Most AL proteins are of the lambda type, but kappa chains may also be the primary protein [1]. AL-amyloidosis is associated with clonal B-cell disorders although most patients do not have overt multiple myeloma. The presentation is dependent on the location of the deposits which are typically systemic but have a tendency to preferentially affect the heart, kidney, gastrointestinal tract, peripheral nerves, skin, and tongue [1].

Although clinical presentation and diagnostic tests such as echocardiogram can suggest amyloidosis, tissue sampling, and identification of amyloid deposits, usually with Congo red staining, are necessary for a definitive diagnosis. We report a case of AL-type amyloidosis with multiple negative biopsy sites including abdominal fat-pad, bone marrow, an osteosclerotic bone lesion, and peripheral arterial and venous vessels. The diagnosis was confirmed by eventual renal biopsy which demonstrated AL-amyloidosis, kappa light chain associated with extensive vascular, interstitial, and glomerular involvement.

## 2. Case Presentation

**Figure 1:**
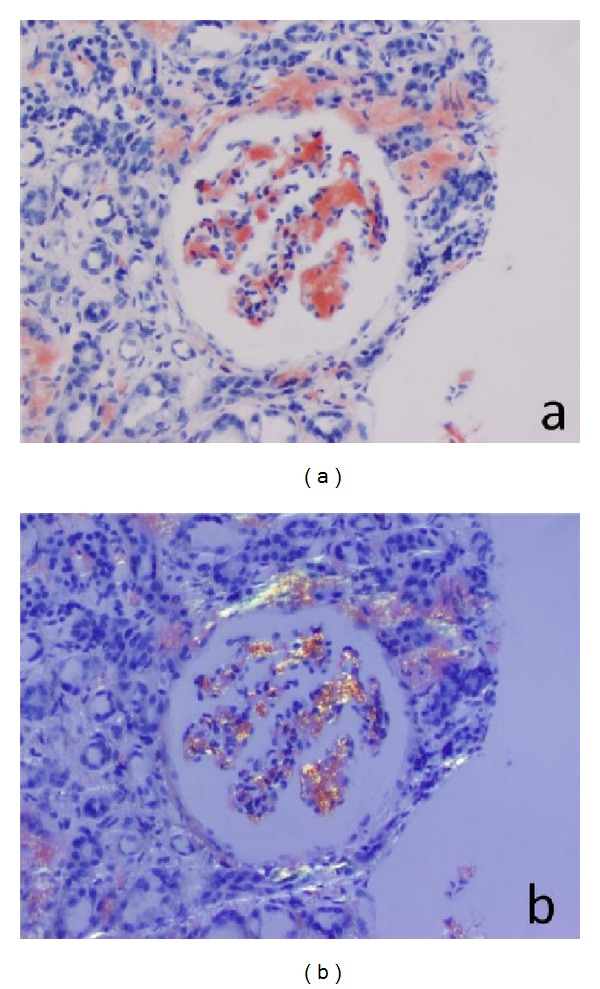
(a) Reddish-orange Congophilic amyloid deposits observed in glomeruli and surrounding interstitium from renal biopsy (b) Congophilic deposits visualized under polarized light demonstrating apple-green birefringence.

A 64-year-old male with a history of CKD Stage 3 with 330 mg/day of proteinuria (eGFR by 4-variable MDRD of 52 mL/min/1.73 m^2^ with a baseline serum creatinine of 1.7 mg/dL), monoclonal gammopathy with elevated kappa light chains on urine protein electrophoresis, and anemia of chronic disease presented over multiple hospitalizations with complaints of peripheral neuropathy, orthostatic hypotension, and unintentional weight loss. The initial investigation included normal levels of vitamin B-12, folate, heavy metals, and was negative for myelin associated glycoprotein antibodies and lyme antibodies. A bone marrow biopsy was completed to rule out progression to multiple myeloma and demonstrated normocellular bone marrow, no clonal plasma cell populations, and an eosinophilic deposit negative for Congo red stain. A 4 mm osteosclerotic lesion was biopsied and demonstrated similar deposits. An abdominal fat-pad biopsy was also negative for Congo red stain. An echocardiogram demonstrated nonspecific findings suggestive of amyloidosis including concentric left and right ventricle hypertrophy, diastolic dysfunction, biatrial enlargement, and tricuspid and mitral valve regurgitation. A renal biopsy was postponed due to concern for a bleeding diathesis as he developed severe right flank ecchymosis after a short coughing bout and subsequently severe thrombocytopenia. A bone marrow biopsy was repeated and amorphous material was suggestive of amyloid deposits but again negative for Congo red stain by two separate pathology laboratories. The patient subsequently developed nonnephrotic range proteinuria of 2.2 gm g/day and acute kidney injury. The acute kidney injury progressed to dialysis-dependent renal failure. During creation of an arteriovenous fistula for dialysis, a segment of the radial artery and brachial vein were submitted for testing and were also negative for Congo red stain. A renal biopsy was performed and was positive for Congo red stain with characteristic apple-green birefringence in the amyloid deposits involving the glomeruli, vessels, and interstitium (Figure 1). Immunofluorescent histology confirmed kappa light chain deposition and electron microscopy demonstrated amyloid deposits characterized by relatively straight, nonbranching, randomly arranged fibrils. Following the completion of the renal biopsy, a final analysis of the second bone marrow biopsy by a third facility recognized as a center of excellence confirmed the presence of AL- (kappa-) type amyloid by liquid chromatography tandem mass spectrometry of peptides microdissected from the bone marrow specimen. The patient was referred to an outside facility for treatment where he received melphalan and dexamethasone. He did not have an adequate hematologic response to therapy and died eight months after AL-amyloidosis was confirmed by renal biopsy.

## 3. Discussion

Amyloidosis may be suspected in patients displaying nonspecific signs such as weakness, weight loss, light headedness, or syncope. Specific organ involvement may result in nephrotic syndrome, renal failure, congestive heart failure, conduction disturbances, or arrhythmias. Abnormal echocardiogram, serum protein electrophoresis (SPEP), or urine protein electrophoresis (UPEP) results may support a diagnosis of amyloidosis. However, histological demonstration of amyloid fibril deposition is required for definitive diagnosis.

Tissue specimen for examination of amyloid deposits can be obtained from almost any source suspected of containing the abnormal protein [3]. Typical sources include abdominal fat-pad, kidney, rectal, gingival mucosa, or bone marrow aspirate. Currently, most samples are obtained from an abdominal fat-pad [2, 5]. Previously, gingival or rectal specimens were commonly obtained [5]. Congo red stain applied to the tissue gives the amyloid protein a salmon-pink color and placed under polarized light the amyloid proteins have an apple-green birefringence. This apple-green birefringence is considered pathognomonic for amyloid fibril deposits. Finally, immunohistochemical staining can be used to confirm amyloid tissue. Treatment decisions depend on determination of the type of amyloid protein. Protein typing can be completed by amino acid sequence analysis and mass spectrometry, immunohistochemical labeling with amyloid-type specific antibodies, enzyme-linked immunosorbent assays, or immunoelectron microscopy [5]. A promising newer technique of laser microdissection and mass spectrometry allows for the isolation and analysis of Congo-red birefringent tissues to determine amyloid type [7].

The tissue source impacts the likelihood of discovering amyloid deposits as described in Table 1. Bone marrow biopsy sensitivity has been estimated at 63% [2]. Kidney, liver, or cardiac biopsies have sensitivity as high as 87–98% but are more invasive [2]. Rectal biopsy sensitivity ranges from 69% to 97% depending upon the quantity of tissue sampled. Rectal biopsy has been largely replaced by abdominal fat-pad aspiration due to clinical advantages [3].

Tissue source also impacts amyloid typing. Immunohistologic analysis cannot be completed from abdominal fat-pad needle aspiration alone and further tissue samples or alternative testing procedures are required for typing [2].

Abdominal fat-pad aspiration is relatively inexpensive, simple, fast, safe, and convenient and is generally regarded as having excellent specificity [2]. However it has limited sensitivity. Bardarov et al. reviewed previous estimates of fat-pad aspiration sensitivity and specificity and found that they ranged from 55 to 80% and 75 to 100%, respectively [4]. Gameren et al. demonstrated sensitivities of 80 to 85% if 30 mg or more of adequate tissue sample was obtained, proper processing and examination completed. The sensitivity increased to > 90% with a more complete examination involving extended visualization time and review by multiple pathologists [2]. Utilization of electron microscopy may also improve sensitivity [5]. Other studies have described sensitivity as low as 19%, but utilized a finer gauge needle, 22-gauge versus 18-gauge, used only light-based microscopy for evaluation of Congo red stains, or very strict criteria to define amyloid-diagnosis [5]. Bardorov also demonstrated that computer-assisted analysis of abdominal fat-pad fine needle aspiration can benefit the specificity and sensitivity of Congo red staining to as high as 75 and 100%, respectively [4]. Alternatively, abdominal fat-pad tissue can be obtained through biopsy of subcutaneous fatty tissue. This larger quantity of tissue is then examined by the same pathologic techniques, and has an estimated sensitivity and specificity of 73% and 90%, respectively [6]. 

Inadequate tissue quantity, inadequate staining, or improper use of polarizing instrument and poor light intensity can all contribute to the number of false negatives and decreased sensitivity [3]. The specific type of Congo red stain, Highman's versus Alkaline, has also been shown to affect sensitivity, 0.11% versus 0.56%, respectively, based on evaluation of stored paraffin-embedded tissue sample analysis of 18 patients with known primary cutaneous amyloidosis [8]. It is unclear if this difference can be generalized to systemic amyloidosis. Tissue fixation prior to staining can also impact sensitivity; fixations in Carnoy's solution or extended time of fixation are associated with decrease sensitivity [3].

Although the specificity of Congo red staining has generally been estimated to be high, the green birefringence of amyloid deposits can be confused with yellow birefringence of collagen, fibrin, or elastin fibers, resulting in false positives [2]. Amyloid deposits can be differentiated from other birefringence materials which maintain the birefringent quality with only a specific horizontal position while amyloid remains birefringent with rotation in the horizontal plane [3]. Exogenous polysaccharides, such as plant cell walls, starch, cotton fibers, and various fungi, can also appear green under polarized light [2].

The nonspecific symptoms of amyloidosis must be confirmed by a histologic tissue biopsy for diagnosis. Multiple options exist for tissue source sampled. Fat-pad biopsies are less invasive and offer reasonable sensitivity. However, care must be taken to ensure adequate sampling, proper staining techniques and careful examination to avoid false negatives. Experienced pathologists or computer-assisted programs may improve sensitivity. Bone marrow samples may be diagnostic for amyloidosis in two thirds of cases. Given the possibility of false negatives, especially with bone marrow biopsies and abdominal fat-pad biopsies, further diagnostic and invasive biopsies should be performed if needed. A kidney biopsy is a more invasive procedure but offers improved sensitivityand is generally safe in experienced hands. Tissue examination and technique are also critical in establishing the diagnosis. If necessary, the tissue should be forwarded to institutions with a high level of amyloid tissue examination experience. 

## Figures and Tables

**Table 1:** Sensitivity and specificity of Congo red staining for amyloidosis related to tissue source.

	Sensitivity (%)	Specificity (%)	Reference
Bone marrow	63		[2]
Kidney, liver, cardiac	87–98		[2]
Rectal	69–97		[3]
Fat-pad needle biopsy	58	100	Guy and Jones cited in [4]
	55	75	Ansari-lari and Ali cited in [4]
	80	100	Hazenberg et al. cited in [4]
	78	93	Dhingra et al. cited in [4]
	80–85		[2]
	>90 increased exam		[2]
	19 (22 gauge)		[5]
Computer assisted	75	100	[4]
Fat-pad biopsy	73	90	[6]
